# Team Science in Precision Medicine: Study of Coleadership and Coauthorship Across Health Organizations

**DOI:** 10.2196/17137

**Published:** 2021-06-14

**Authors:** Ning An, John Mattison, Xinyu Chen, Gil Alterovitz

**Affiliations:** 1 Key Laboratory of Knowledge Engineering with Big Data of the Ministry of Education, Intelligent Interconnected Systems Laboratory of Anhui Province School of Computer Science and Information Engineering Hefei University of Technology Hefei China; 2 Kaiser Permanente Arsenal Capital Partners New York City, NY United States; 3 Department of Electrical and Computer Engineering University of California San Diego La Jolla, CA United States; 4 Brigham and Women's Hospital Harvard Medical School Boston, MA United States

**Keywords:** precision medicine, team science

## Abstract

**Background:**

Interdisciplinary collaborations bring lots of benefits to researchers in multiple areas, including precision medicine.

**Objective:**

This viewpoint aims at studying how cross-institution team science would affect the development of precision medicine.

**Methods:**

Publications of organizations on the eHealth Catalogue of Activities were collected in 2015 and 2017. The significance of the correlation between coleadership and coauthorship among different organizations was calculated using the Pearson chi-square test of independence. Other nonparametric tests examined whether organizations with coleaders publish more and better papers than organizations without coleaders.

**Results:**

A total of 374 publications from 69 organizations were analyzed in 2015, and 7064 papers from 87 organizations were analyzed in 2017. Organizations with coleadership published more papers (*P*<.001, 2015 and 2017), which received higher citations (*Z*=–13.547, *P*<.001, 2017), compared to those without coleadership. Organizations with coleaders tended to publish papers together (*P*<.001, 2015 and 2017).

**Conclusions:**

Our findings suggest that organizations in the field of precision medicine could greatly benefit from institutional-level team science. As a result, stronger collaboration is recommended.

## Introduction

The concept of a meta-topical brainforest is proposed, to reflect a link between collaborative research and complex ecosystems. Tropical rainforests leverage a diversity of species to capture and convert solar energy into carbon-based life, and research teams can harvest a similar benefit from a diversity of data, tools, and thought paradigms.

According to the National Institutes of Health, team science is “a collaborative and often cross-disciplinary approach to scientific inquiry that draws researchers who otherwise work independently or as coinvestigators on smaller-scale projects into collaborative centers and groups” [1]. Thus, team science occurs when artificial boundaries such as departments and institutions are crossed, allowing collaboration in integrated networks. Over the past two decades, the concept has received increasing attention to better understand and address global challenges [2-5]. In 2007, Wuchty et al [6] examined 19.9 million research articles in the Institute for Scientific Information Web of Science database and 2.1 million patent records on multiple topics. They concluded that a team-authored paper has increased probability of being highly cited. The systems being formed through interdisciplinary collaborations help teams reach achievements that individual researchers are less likely to accomplish.

Kohane [7] pointed out that precision medicine in particular requires a higher level of coordination between various agencies and suggests the boundaries between research projects and clinical care institutions should be blurred to link gathered data. The exponential growth and causal interdependencies of “-omics” fields dictate that expertise across disciplines is essential to making meaningful and durable contributions to the understanding of human biology.

This brief viewpoint aims to explore the impact of cross-institution team science on the development of precision medicine. We hypothesized that international organizations with coleaders tend to publish more impactful papers than organizations without coleaders. Using the Pearson chi-square test and the Mann-Whitney *U* test, we validated our hypothesis.

## Methods

Information was collected from the eHealth Catalogue of Activities developed by the nonprofit Global Alliance for Genomics and Health in 2015 [8]. The catalog lists international genomic and clinical data-sharing initiatives, and the eHealth Task Team updated the catalog through 2017. The data on the executive leadership team and publications were obtained from the websites of these organizations. If such information was not found, additional data were acquired by directly contacting the organizations or searching on Google Scholar. The impact of papers was evaluated by their number of citations, a criterion of research quality [6].

In this paper, coleadership means that a person holds a leadership position in different organizations concurrently. If two papers from separate organizations have at least one author in common, these two organizations are regarded as having a coauthor relationship.

Nonparametric tests were performed to verify the hypothesis. We used SPSS (version 22.0; IBM Corp) and R to perform two-tailed tests with an α level of .05. The significance of the correlation between the nominal variables coleadership and coauthorship was examined using the Pearson chi-square test of independence and expressed in a contingency table. The Pearson chi-square test of goodness of fit was adopted to evaluate whether organizations with coleaders had a greater number of publications than organizations without coleaders, and the Mann-Whitney *U* test was used to examine whether the former organizations published papers that received more citations than the latter.

## Results

### Overview

We analyzed data from 69 organizations in the catalog and found 16 pairs with coleader relationships in 2015. Among the 374 publications from these organizations at that time, 13 pairs had coauthors. By 2017, the number of institutions in the catalog increased to 87, and there were 37 pairs with coleadership, corresponding to 30 organizations. Information on 7064 papers was collected, showing that 55 organizations had coauthored publications, with 436 papers in total.

### Number of Publications

The chi-square goodness of fit test suggests that the number of papers being published is strongly correlated with the category of the organization—organizations in a coleadership network or organizations without coleadership (*P*<.001, 2015 and 2017).

### Quality of Publications

The citation number of each paper was obtained from Google Scholar. The results of the Mann-Whitney *U* test indicated that the number of citations received by publications of organizations with and without coleaders differed significantly (*Z*=–13.547, *P*<.001, 2017). Papers from the former organizations had a higher mean rank (3603.35 for the group of papers whose authors are in the coleadership network, and 2702.67 for the other group), which means that the organizations with coleaders tended to have a greater number of highly cited papers.

### Relationship Between Coleader and Coauthor

In the chi-square test of independence, the total sample size is the number of lines in a fully connected diagram. The results indicate that in both 2015 and 2017, organizations with coleaders tended to publish papers together, suggesting that coleadership will lead to coauthorship (*P*<.001, 2015 and 2017).

## Discussion

We studied how precision medicine can be influenced by institutional-level team science by analyzing coleadership and coauthorship across health organizations. From 2015 to 2017, the number of health organizations grew from 69 to 87, and their publications increased. Concurrent positions held by leaders may incentivize researchers to work for multiple organizations; thus, the researchers will be very likely to have a coauthored paper (*P*<.001, both 2015 and 2017). Moreover, the publications from organizations with coleaders are more frequently cited, indicating a relatively high quality (*Z*=–13.547, *P*<.001). These results suggest that collaborations among health institutions are becoming stronger, which promotes their working efficiency.

**Figure 1:**
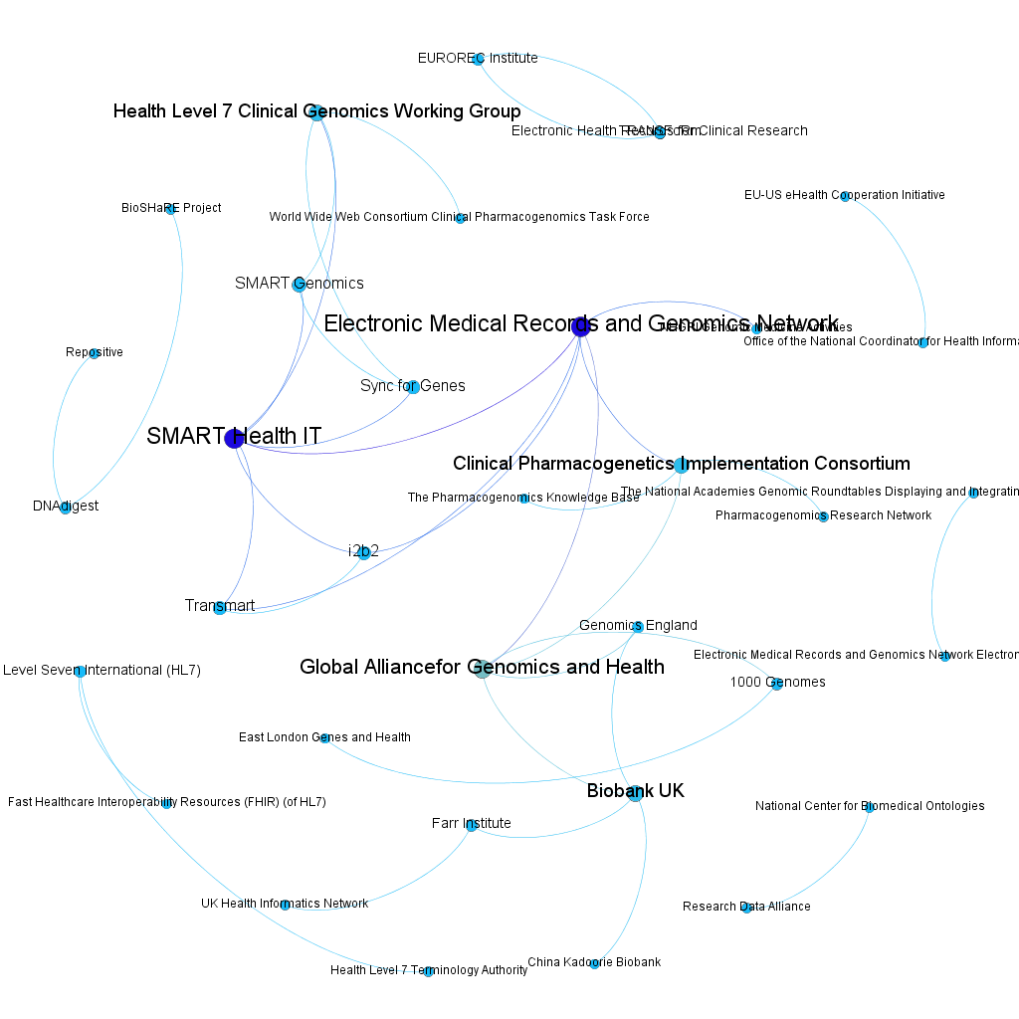
The coleader relationship network in 2017, with nodes representing organizations and lines representing concurrent coleadership.

**Figure 2:**
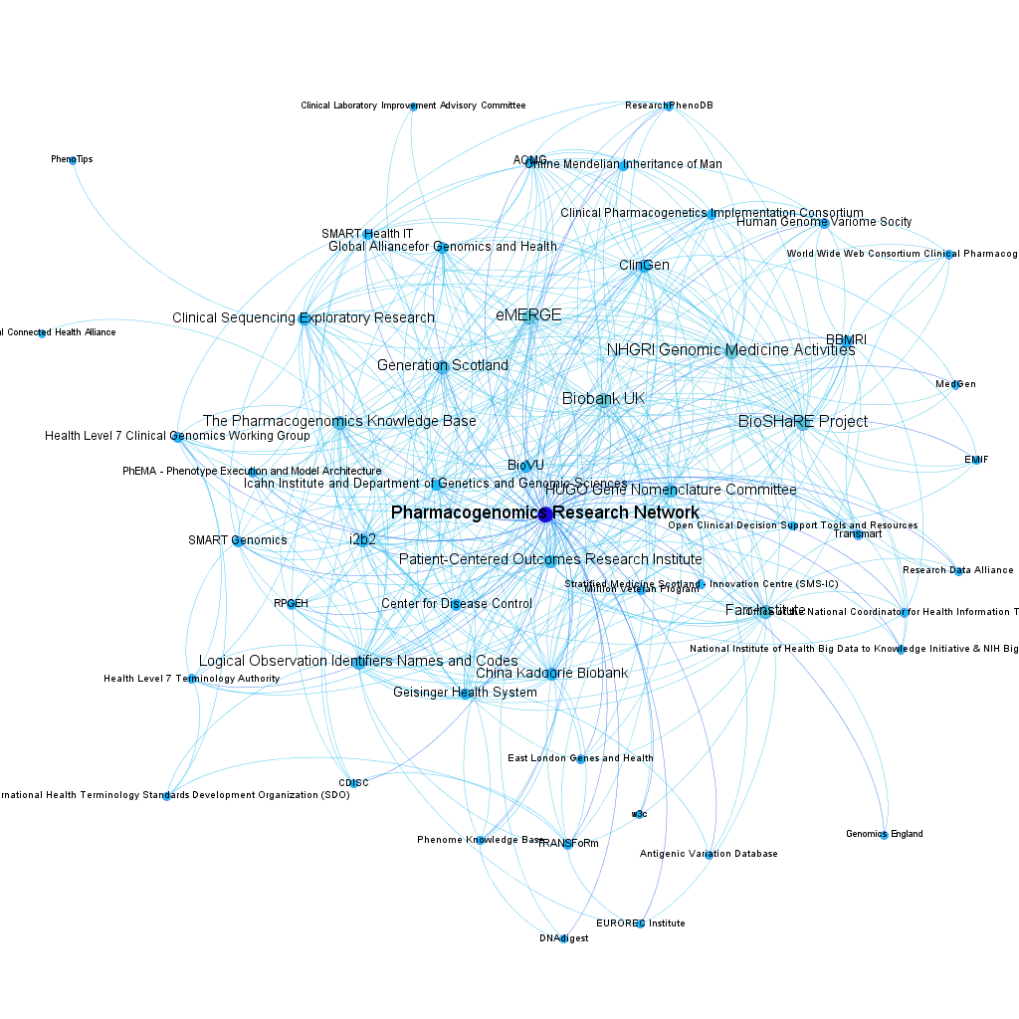
The coauthor relationship in 2017, with nodes representing organizations and lines connecting organizations by coauthored publications. Nodes darken with more connected lines.

These results illustrate the concept of meta-topical brainforests in precision medicine (Figures 1-2) and may have broader implications: cross-enterprise cooperation plays an essential role in solving complex issues. As a field-crossing example, Sovacool [9] suggested researchers should incorporate expertise and data from indigenous groups to address global environmental challenges.

One hopes the analogy persists and the extraordinary natural future-proofing mechanisms in rainforests coincide with similar continued diversification in research networks and widely impactful publications.
